# Toward a Genomics-Driven Hepatology: Liver Biology, Precision Diagnosis, and the Rise in Genetic Therapies

**DOI:** 10.3390/pharmaceutics18040455

**Published:** 2026-04-08

**Authors:** Sri Harsha Boppana, Naveena Luke, Sravani Karuchola, Jahnavi Udaikumar, Cyrus David Mintz

**Affiliations:** 1 Department of Internal Medicine, Nassau University Medical Center, 2201 Hempstead Tpke, East Meadow, NY 11554, USA; 2 Department of Internal Medicine, NYU Grossman School of Medicine, New York, NY 10016, USA; 3 HCA Healthcare Clear Lake, Webster, TX 77598, USA; sravani.karuchola@gmail.com; 4 Department of Anesthesiology & Critical Care Medicine, Johns Hopkins School of Medicine, 1800 Orleans St, Baltimore, MD 21287, USA

**Keywords:** hepatology, genomic diagnostics, genetic therapies, liver sinusoidal endothelial cells, Kupffer cells, immune microenvironment, precision medicine, clinical implementation

## Abstract

The liver’s anatomic position and immune specialization make it both a major target and a major filter for systemically delivered therapeutics. Because portal venous inflow exposes the liver early to gut-derived molecules and exogenous compounds, many intravenously administered agents, including gene-based medicines and their viral and non-viral delivery systems, preferentially enter and accumulate in hepatic tissue. This review synthesizes how core liver physiology and immunobiology influence the performance, safety, and clinical translation of genomic medicines in hepatology, and outlines near-term practice and research shifts likely to define a genomics-driven future in liver disease care. We review the hepatic microarchitecture relevant to therapeutic trafficking, including sinusoidal transit, the space of Disse, hepatocyte uptake, and hepatobiliary elimination, and highlight the gatekeeping roles of liver sinusoidal endothelial cells and Kupffer cells in clearing particulate material and shaping inflammatory signaling. We then discuss how these same features create both opportunities, such as efficient hepatic targeting, and constraints, including innate immune activation, vector clearance, and variable intrahepatic distribution, for DNA- and RNA-based platforms. Finally, we propose five actionable developments poised to move genomics from a niche tool to a routine component of hepatology practice: earlier genomic testing in unexplained liver disease, multidisciplinary hepatology genome rounds, a centralized liver-specific gene resource, genetics-aware clinical trial design, and expansion of genetic therapies. Integrating liver biology with genomic medicine is essential to improve diagnostic yield, personalize therapy, and accelerate translation of gene-based treatments while mitigating immunologic and delivery-related barriers.

## 1. Introduction

Genomics-driven hepatology refers to the systematic integration of genomic information, including next-generation sequencing (NGS), genome-wide association data, polygenic risk scores, and the interpretation of both germline and somatic variants, into the routine evaluation and management of liver disease [1]. It extends beyond simply identifying monogenic disorders; it encompasses the use of genetic architecture to clarify disease mechanisms, predict progression, stratify risk, and guide therapeutic selection across both rare and common hepatic conditions [2,3,4,5].

At its core, genomics-driven hepatology represents a shift from phenotype-based classification to mechanism-based diagnosis. By integrating genotypic data with clinical presentation, histopathology, laboratory parameters, and environmental exposures, clinicians can move toward precision hepatology where care is individualized according to the molecular drivers of disease [6,7]. This approach has implications not only for diagnosing rare inherited cholestatic or metabolic disorders, but also for understanding susceptibility and treatment response in highly prevalent conditions such as metabolic dysfunction–associated steatotic liver disease (*MASLD*), alcohol-associated liver disease, autoimmune hepatitis, and hepatocellular carcinoma.

Genomics-driven hepatology also incorporates emerging tools such as transcriptomic profiling, somatic mutation analysis in liver tumors, and reanalysis of sequencing data as variant interpretation evolves. Importantly, it emphasizes clinical utility—identifying when genomic information meaningfully alters patient management, such as directing targeted therapy, prompting family cascade testing, influencing transplant candidacy decisions, or reducing unnecessary invasive procedures. In this framework, genomics becomes not a research adjunct, but a clinical instrument that refines diagnosis, improves prognostication, and enables therapeutic innovation. In addition to disease-focused genomic testing, pharmacogenomic profiling, including genotyping of cytochrome P450 (CYP) enzymes, may also have clinical relevance in hepatology. Although CYP testing does not usually establish the primary etiology of liver disease, it can be important for individualized therapy by identifying patients who are poor, intermediate, normal, or ultrarapid metabolizers and therefore may be at increased risk for altered drug efficacy, toxicity, or hepatotoxicity.

This review is organized around a central premise: the liver’s unique biologic architecture creates both opportunities and barriers for genomic medicine implementation. First, we outline the aspects of liver physiology and immunobiology that make the liver uniquely responsive, but also uniquely vulnerable, to gene-based and molecular interventions. Second, we describe five near-term developments that are likely to shape a genomics-driven future in hepatology, including earlier genomic testing, structured hepatology genome rounds, improved liver-specific gene-resource infrastructure, genomics-informed trial design, and the emergence of genetic medicines. Finally, we discuss how this framework may evolve beyond genomics alone toward multiomics integration and real-world data platforms that further refine diagnosis, risk stratification, and therapy.

## 2. Liver Physiology and Immunology

### 2.1. Core Metabolic and Clearance Functions

The liver plays a central role in nutrient metabolism, including protein, lipid, and carbohydrate homeostasis, and in the clearance of metabolic waste products [8]. A core function is to regulate the entry of substances into the systemic circulation by screening and processing compounds absorbed from the gastrointestinal tract before they move beyond the liver [9].

### 2.2. Dual Blood Supply and First-Pass Exposure to Gut-Derived Substances

A distinctive feature of the liver is its dual blood supply. The portal vein delivers nutrient-rich venous blood from the digestive organs and accounts for approximately two-thirds of hepatic blood flow, whereas the hepatic artery provides the remaining flow and delivers highly oxygenated blood. Blood exits the liver through the hepatic veins and drains into the inferior vena cava. As the first organ to receive portal venous outflow from the intestines, the liver is exposed early and extensively to gut-derived substances [10,11]. This anatomy, together with the liver’s detoxification role, means that many exogenous compounds reach the liver in high concentrations. In the context of genomic medicine, this is particularly relevant because the liver is a major site for recognizing and clearing unencapsulated therapeutics as well as viral and non-viral delivery vectors that enter the circulation. As a result, many intravenously administered agents, including gene-based therapies and their delivery systems, preferentially enter and accumulate in the liver [12].

### 2.3. Structural Organization: Hepatic Lobule and Microanatomy

The hepatic lobule is considered the main functional unit of the liver [13]. It is organized around plates of hepatocytes and includes the portal triads, a central vein, hepatic sinusoids running from the portal regions toward the central vein, Kupffer cells (resident hepatic macrophages), bile canaliculi, and the space of Disse (the narrow compartment between sinusoidal blood and hepatocytes). Each portal triad typically contains a small portal vein branch, a hepatic artery branch, and a bile duct. As blood traverses the sinusoids and the space of Disse en route to the central vein, hepatocytes extract, process, store, and transform nutrients and other circulating molecules.

### 2.4. Major Hepatic Cell Populations and Extracellular Matrix

Hepatocytes account for approximately 80% of the liver’s parenchymal mass, whereas non-parenchymal cells account for approximately 20%. These non-parenchymal populations include cholangiocytes, hepatic stellate cells, Kupffer cells, and liver sinusoidal endothelial cells. The remaining liver mass includes extracellular matrix components.

### 2.5. Liver Sinusoidal Endothelial Cells, Kupffer Cells, and Hepatic Stellate Cells: Key Roles

LSECs are highly efficient at clearing viruses, bacteriophages, and particulate material bearing exogenous ligands from the bloodstream, largely because of their extensive surface area and broad receptor repertoire [14]. Unlike typical vascular endothelium, LSECs lack a basal lamina and contain numerous fenestrae (approximately 150–200 nm in diameter) that occupy about 6–8% of the endothelial surface [15]. Kupffer cells, together with LSECs, form a major part of the hepatic reticuloendothelial system, capturing and eliminating pathogens, antigens, and damage-associated molecular patterns, while also producing inflammatory cytokines. Hepatic stellate cells primarily store vitamin A under normal conditions; however, with chronic liver injury they can differentiate into myofibroblast-like cells that synthesize interstitial matrix components, driving fibrotic scar formation.

### 2.6. LSECs in Liver Disease and Regeneration

The role of LSECs in liver disease is complex and has been described in conflicting ways. Some evidence suggests they help maintain HSC quiescence, which limits intrahepatic vasoconstriction and slows fibrogenesis [16,17]. Other studies support a central role for LSECs in initiating and promoting chronic liver disease progression [18,19,20]. LSECs also contribute to liver regeneration after acute injury or partial hepatectomy, in part through signaling that supports hepatocyte proliferation. Overall, the major hepatic cell types are tightly interconnected and collectively shape both normal liver physiology and disease responses.

### 2.7. Hepatobiliary Elimination Pathway

Hepatobiliary elimination generally follows a stepwise route: circulating substances move from the bloodstream into (1) the liver sinusoid, then (2) the space of Disse, then (3) hepatocytes, followed by secretion into (4) bile ducts, passage into (5) the intestines, and finally (6) excretion from the body [21,22]. Understanding how substances move through these compartments at the cellular level is important for designing effective therapeutics and delivery platforms.

### 2.8. Hepatic Immune Identity: Lymphoid Organ Features and Innate Bias

Immunologically, the liver functions as a lymphoid organ with distinctive features, including a strong innate immune bias. Its immune cell composition differs from peripheral lymphoid organs (such as spleen and lymph nodes) and includes Kupffer cells, LSECs, neutrophils, natural killer cells, innate lymphoid cells, natural killer T cells, γδ T cells, and mucosal-associated invariant T cells, which together support immune surveillance and liver homeostasis [23,24,25].

### 2.9. Kupffer Cells, Inflammatory Cytokines, and T-Cell–Mediated Injury

Kupffer cells are particularly important in initiating hepatic inflammation [26]. When activated, they produce cytokines such as IL-12, IL-6, IL-1β, and IL-8, along with TNF-α and transforming growth factor beta, which are key mediators linked to fibrogenic signaling [27]. TNF-α also promotes T-cell activation, which can amplify cytotoxic immune responses. Accumulation of highly activated intrahepatic CD8+ T cells is a common feature across many liver diseases and is considered an important component of their pathogenesis [28].

### 2.10. B Cells, Antibody-Dependent Mechanisms, and Tissue Repair Crosstalk

The role of B cells in the adult liver is less well characterized, but available evidence suggests they may contribute to fibrotic progression in some settings. Antibody-dependent mechanisms have been implicated in liver injury, and antibody production has been shown to play an important role in alcohol-associated liver damage [29]. Beyond host defense, hepatic immune networks also coordinate tissue repair following cell injury and loss. Although the liver’s immune environment is partially compartmentalized from systemic immunity, it interacts closely with the intestines and with peripheral immune organs through continuous antigen and cytokine trafficking [30]. Because immune responses are integral to essentially all liver diseases, understanding hepatic immunobiology and the ways it can be modulated is a key foundation for developing effective therapies. Taken together, these physiologic and immunologic features help explain why the liver occupies a central position in genomic medicine. The fenestrated sinusoidal endothelium and the absence of a classic basement membrane facilitate access of circulating agents to the hepatocyte surface, making the liver an attractive target for nucleic acid therapeutics and vector-based delivery platforms. At the same time, liver sinusoidal endothelial cells and Kupffer cells act as major biologic filters that can sequester, clear, or immunologically react to viral vectors, lipid nanoparticles, and other exogenous cargos before they achieve uniform hepatocyte delivery. These same features have direct implications for implementation: molecular diagnosis is especially important when considering gene-based therapy because treatment selection, expected efficacy, safety, and eligibility for clinical trials depend not only on the disease-causing genotype but also on whether the therapeutic platform can successfully navigate the hepatic microenvironment. Accordingly, liver biology should be viewed as the mechanistic bridge between genomic diagnosis and the clinical translation of genetic medicines in hepatology.

## 3. Where Hepatology Is Headed: A Genomics-Driven Future

The National Human Genome Research Institute recently laid out a strategic vision for how genomics could reshape human health over the next decade, highlighting 10 ambitious predictions [30]. Their outlook includes genomic data becoming routine in everyday clinical care, a clearer understanding of the function of essentially all ~20,000 human genes, and a much more complete grasp of which genetic variants truly matter clinically [30]. In hepatology, we appear to be approaching a turning point where genomic medicine shifts from a specialized tool to a standard part of practice. Building on that broader vision, we propose five near-term developments that could meaningfully change how liver disease is evaluated, studied, and treated.

### 3.1. Development 1: Earlier Genomic Testing for Unexplained Liver Disease

**Figure 1:**
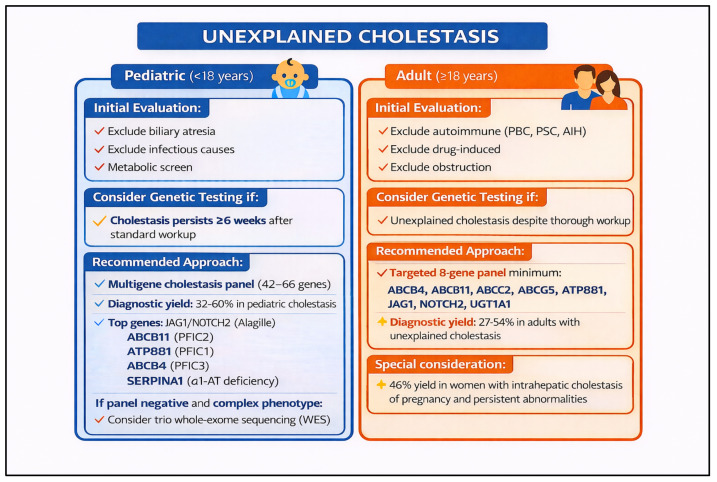
Diagnostic yield of next-generation sequencing (NGS) panel testing in neonatal, infantile, and pediatric cholestasis. Bar graph illustrating the proportion of genetically diagnosed cases across studies using targeted NGS gene panels in cholestatic neonates, infants, children, and young adults. Studies employed panels ranging from disease-specific targeted sets to comprehensive multi-gene cholestasis panels (including a 66-gene panel). Diagnostic yields varied across cohorts reflecting differences in panel size, patient age at presentation, and phenotypic severity.

We anticipate that genomic testing will increasingly be used early in the evaluation of patients with unexplained liver disease, with the potential to identify a genetic diagnosis in at least 20% of such cases. When a genetic etiology is confirmed, the downstream effects can be substantial—guiding targeted treatment, informing preventive screening strategies, and supporting family counseling and cascade testing [31]. Many of these patients currently experience a prolonged “diagnostic odyssey,” often involving years of referrals, repeated testing, and invasive procedures. Earlier use of genomic analysis could reduce that burden for patients and lower overall health care utilization [32], and it may decrease the need for liver biopsy in some pediatric evaluations. The diagnostic value of NGS-based panel testing has been most clearly demonstrated in pediatric cholestatic liver disease. Studies using targeted gene panels in neonates and infants with cholestasis of unclear etiology have reported diagnostic yields ranging from approximately 30% to over 50%, with higher yields observed when broader panels and earlier testing are employed (Figure 1) [33,34,35]. A large multicenter study using a comprehensive 66-gene cholestasis sequencing panel in over 2000 cholestatic infants, children, and young adults confirmed both the feasibility and clinical utility of panel-based approaches at scale. Importantly, a genetic diagnosis in these cohorts frequently altered clinical management—prompting disease-specific treatments, informing prognosis, and avoiding unnecessary invasive workup [34]. These findings support the earlier and more systematic integration of NGS panel testing into the evaluation of unexplained pediatric cholestasis, and underscore the need for analogous frameworks in adult hepatology [36,37].

**Figure 2:**
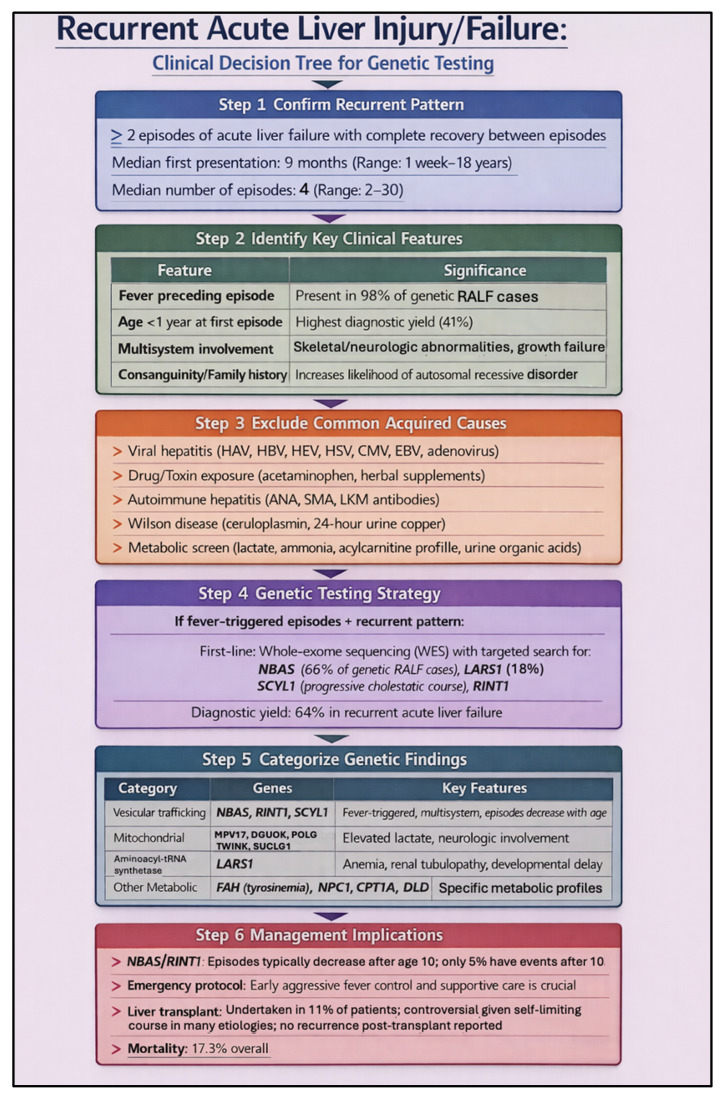
Genetic Testing Algorithm for Pediatric Acute Liver Failure (PALF) of Indeterminate Origin. This algorithm outlines a stepwise approach to genetic evaluation in children presenting with acute liver failure without an identified etiology after standard workup. Initial assessment incorporates clinical phenotyping, metabolic screening, and family history to stratify patients by likelihood of an underlying genetic cause. Subsequent tiers of genomic testing—ranging from targeted gene panels to whole-exome or whole-genome sequencing—are guided by phenotypic features, recurrence, and initial diagnostic yield. Genetic diagnoses identified through this approach include mitochondrial disorders, metabolic enzyme deficiencies, and immune dysregulation syndromes, among others. Early genetic diagnosis informs prognosis, guides acute management, directs transplant decision-making, and facilitates family counseling.

Pediatric acute liver failure (PALF) of indeterminate origin represents one of the most urgent and diagnostically challenging scenarios in which early genomic testing can meaningfully alter clinical management [38]. A significant proportion of PALF cases remain without an identified etiology even after conventional evaluation, and emerging evidence suggests that genetic causes account for a substantial subset of these cases, particularly in those with recurrent episodes or a suggestive family history [38,39,40]. Lenz et al. and Peters et al. demonstrated that genomic analysis of PALF of indeterminate origin identified a genetic diagnosis in a meaningful proportion of patients, revealing conditions such as mitochondrial respiratory chain disorders, vesicular trafficking disorders, inborn errors of metabolism, and immune dysregulation syndromes that would not have been detectable through standard biochemical workup alone [38,39]. Systematic review data further highlight that recurrent PALF is especially enriched for underlying genetic etiologies, with long-term outcomes closely tied to whether a specific molecular diagnosis is established [39,40,41]. Hegarty and Thompson have similarly cataloged the expanding spectrum of genetic causes of acute liver failure in children, underscoring the clinical value of a broad sequencing approach early in the disease course [41,42]. Identifying a genetic etiology in PALF not only clarifies prognosis but can directly influence acute therapeutic decisions, guide transplant listing and candidacy, enable targeted treatment where available, and support cascade testing in families. Figure 2 illustrates a structured algorithmic approach to genetic evaluation in this population, emphasizing early deployment of genomic tools and phenotype-guided test selection.

#### 3.1.1. Implementation Needs: Best-Practice Guidance, Funding Justification, and Decision Support

To make this shift realistic, hepatology-specific best practice guidance is needed for when and how to implement genomic testing in both adults and children. Clear guidance also helps clinicians justify testing to payers, since insurance coverage remains a major barrier—particularly in adult hepatology clinics. A practical approach would be to leverage electronic medical record decision-support tools: once a clinician documents a complete conventional workup and the cause remains unclear, the system could prompt genomic testing as a logical next step in management.

**Figure 3:**
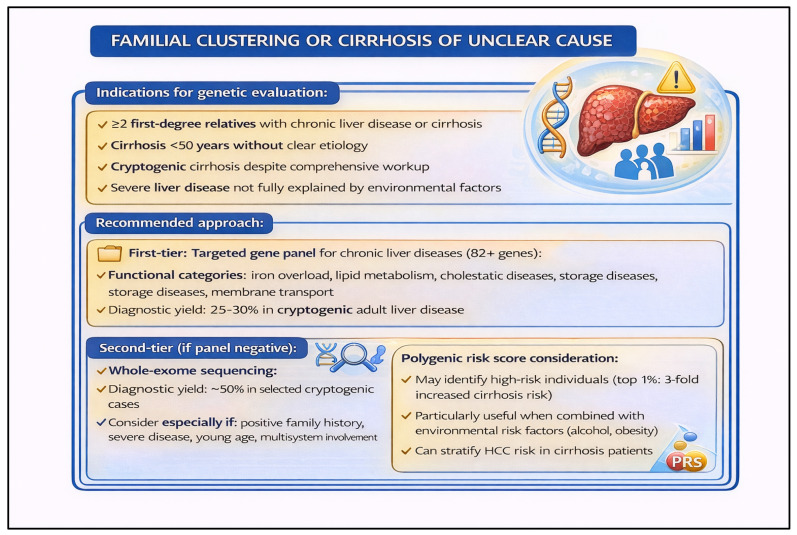
Genomic Evaluation Framework for Chronic Liver Disease with Suspected Genetic Etiology. This figure outlines a structured approach to genetic testing in patients with chronic liver disease of unclear or suspected genetic origin, including those with metabolic dysfunction–associated steatotic liver disease (*MASLD*), cryptogenic cirrhosis, or unexplained elevated liver enzymes. Patients are stratified by clinical phenotype and risk profile to guide selection between targeted gene panels, exome sequencing, and polygenic risk score analysis. Diagnostic yields and key gene targets (including *PNPLA3*, *TM6SF2*, *HSD17B13*, *MBOAT7*, and *SERPINA1*) are highlighted across clinical subgroups. Integration of common and rare variant data supports both diagnostic clarification and risk stratification for disease progression, including cirrhosis.

The approach to genetic testing in chronic liver disease requires careful phenotype-driven stratification. For patients presenting with unexplained chronic liver disease, elevated liver enzymes without a clear etiology, or features suggestive of *MASLD* in the absence of traditional metabolic risk factors (so-called “lean NAFLD”), genomic analysis has demonstrated meaningful diagnostic and prognostic utility [43,44]. Common variants in genes such as *PNPLA3*, *TM6SF2*, *HSD17B13*, and *MBOAT7* have been associated with susceptibility to steatosis, fibrosis progression, and cirrhosis risk across diverse populations [45,46]. Importantly, rare, high-impact variants identified through targeted gene panels or exome sequencing can unmask monogenic contributors that may otherwise be misclassified as polygenic or environmental disease [46,47]. Combining panel-based next-generation sequencing with exome sequencing has been shown to improve diagnostic yield in both pediatric and adult populations with suspected genetic liver disease [47,48]. Polygenic risk scores incorporating multiple common variants offer a complementary strategy for risk stratification, particularly for predicting progression to advanced fibrosis or cirrhosis in patients who do not carry a single high-impact variant [48,49]. Figure 3 illustrates a practical framework for applying these genomic strategies across common clinical presentations of chronic liver disease with a suspected genetic component.

#### 3.1.2. Clinical Scenario-Based Decision Tree for Genetic Testing in Liver Disease

**Figure 4:**
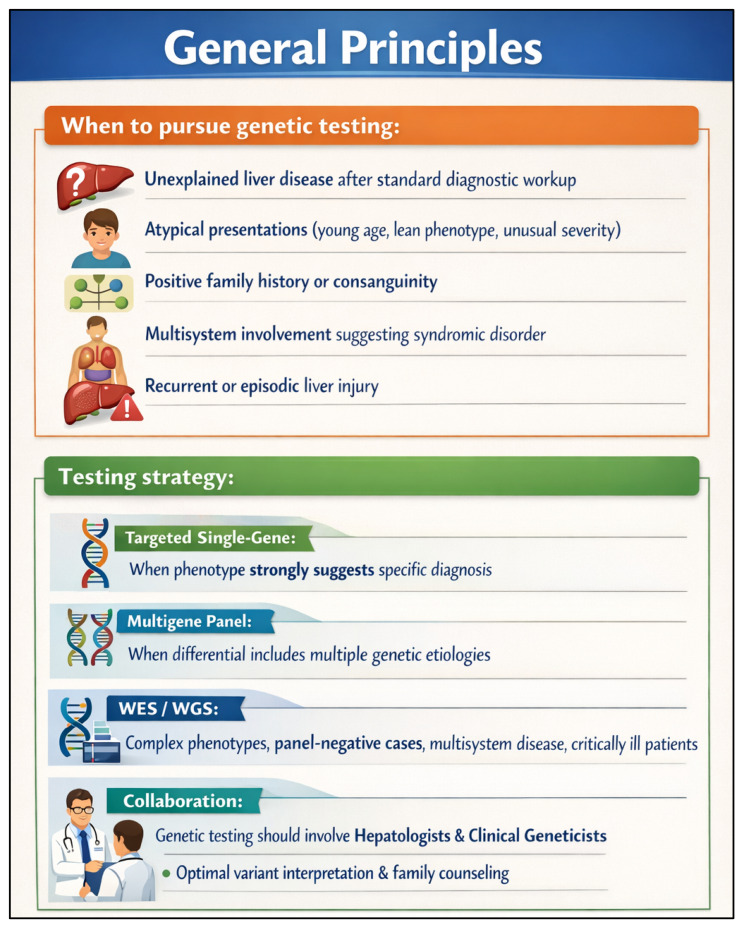
Clinical Scenario-Based Decision Tree for Genetic Testing in Liver Disease. A structured, phenotype-driven framework guiding clinicians through five common clinical scenarios encountered in genomics-driven hepatology: (1) unexplained cholestasis in adults and adolescents, (2) neonatal and infantile cholestasis, (3) pediatric acute liver failure of indeterminate origin, (4) lean or atypical NAFLD/*MASLD*, and (5) cryptogenic or idiopathic liver disease in adults. For each scenario, the decision tree identifies recommended first-line genomic testing strategies (targeted gene panel, exome sequencing, or polygenic risk score analysis), key genes of interest, and approximate diagnostic yield ranges derived from published cohort data. In adults and adolescents with unexplained cholestasis, targeted panel sequencing of genes including *ABCB4*, *ATP8B1*, and *ABCB11* yields a genetic diagnosis in approximately 15–30% of cases. In neonatal and infantile cholestasis, comprehensive multi-gene panels (including 66-gene platforms) achieve diagnostic rates of approximately 30–50%, with higher yields when broader panels and earlier testing are employed. In paediatric acute liver failure of indeterminate origin, genomic evaluation identifies a pathogenic or likely pathogenic variant in a meaningful subset of cases (reported ranges ~15–40%), revealing conditions such as mitochondrial disorders, inborn errors of metabolism, and immune dysregulation syndromes. In lean or atypical *MASLD*, panel or exome sequencing can reveal high-impact rare variants (e.g., *PNPLA3*, *TM6SF2*, *HSD17B13*, *MBOAT7*) that explain an otherwise puzzling phenotype. In adults with cryptogenic or idiopathic liver disease after standard workup, clinical exome or genome sequencing offers an additional diagnostic tier with reported yields of approximately 15–25%. The framework is intended to assist clinicians in determining when and how to initiate genetic evaluation, and to support downstream management including targeted therapy, cascade testing, and avoidance of unnecessary invasive procedures.

To reduce variation in testing practice, we developed a phenotype-driven decision tree spanning five common clinical scenarios in hepatology: unexplained cholestasis in adults and adolescents, neonatal and infantile cholestasis, pediatric acute liver failure of indeterminate origin, lean or atypical *MASLD*, and cryptogenic or idiopathic liver disease in adults. Rather than duplicating the full logic of the algorithm in the text, Figure 4 is intended to provide the operational pathway, while the accompanying discussion highlights the evidence base and the clinical rationale for earlier genomic evaluation in each setting. This decision tree provides a structured approach to genetic testing across five common clinical scenarios in genomics-driven hepatology. The framework emphasizes phenotype-driven testing strategies, with clear diagnostic yields and gene targets for each scenario, helping clinicians determine when and how to pursue genetic evaluation. Figure 4 presents a phenotype-driven decision tree designed to help clinicians determine when and how to pursue genetic testing across five common clinical scenarios in hepatology. In adults and adolescents with unexplained cholestasis, targeted gene panel sequencing has demonstrated meaningful diagnostic yields and can identify variants in genes such as *ABCB4*, *ATP8B1*, and *ABCB11* that directly inform management [36]. In neonatal and infantile cholestasis, next-generation sequencing panels encompassing a broad range of cholestatic disease genes have been shown to achieve diagnostic rates exceeding 40% in some cohorts, supporting their early integration into the evaluation of affected infants [37,50]. For pediatric acute liver failure of indeterminate origin, emerging evidence points to a substantial subset of cases harboring pathogenic or likely pathogenic variants, making rapid genomic evaluation increasingly relevant in this high-acuity setting [37]. In lean or atypical presentations of *MASLD*, genomic analysis can uncover rare, high-impact variants that explain an otherwise puzzling phenotype and may redirect clinical management [50]. Finally, in adults with cryptogenic or idiopathic liver disease who have completed a standard workup without a diagnosis, clinical exome or genome sequencing offers an additional diagnostic tier with reported yields ranging from approximately 15% to over 25% depending on cohort selection and sequencing strategy [37,50]. Across all five scenarios, earlier genomic testing has the potential to shorten the diagnostic odyssey, reduce unnecessary procedures, and open pathways to targeted or mechanism-based therapy.

**Figure 5:**
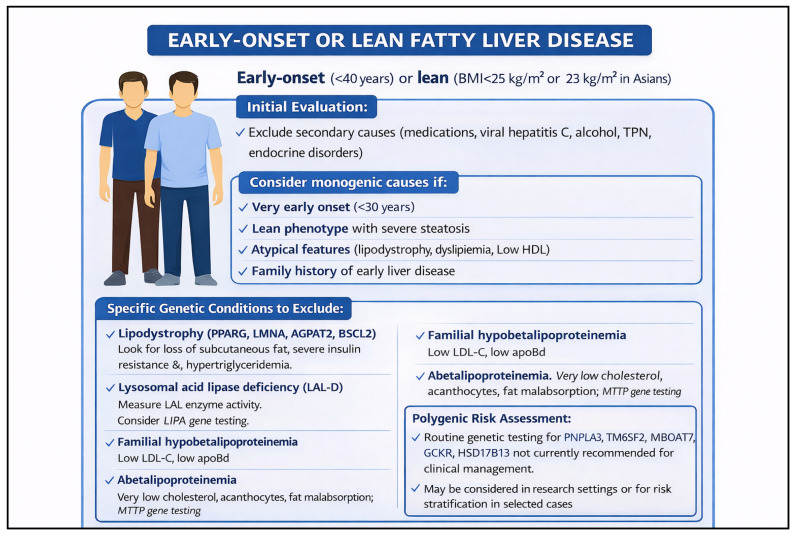
Genetic Testing Algorithm for Metabolic Dysfunction-Associated Steatotic Liver Disease (*MASLD*) in Lean and Atypical Presentations. This figure illustrates a stepwise clinical decision framework for incorporating genetic evaluation into the workup of patients presenting with *MASLD* or unexplained hepatic steatosis, particularly in lean individuals or those lacking classical metabolic risk factors. The algorithm integrates standard clinical assessment with genotype-guided risk stratification, highlighting key variants (e.g., *PNPLA3*, *TM6SF2*, *HSD17B13*) and their implications for disease progression and individualized management.

A distinct and increasingly recognized challenge involves patients with hepatic steatosis who lack classical metabolic risk factors, as well as those whose disease severity appears disproportionate to their metabolic burden. In this setting, Figure 5 provides a practical phenotype-driven algorithm, while the text below emphasizes why this subgroup is particularly well suited to genomics-integrated evaluation. In these populations, genetic evaluation is particularly informative. Current clinical practice guidelines recommend a thorough assessment of secondary causes of steatosis before attributing disease to metabolic dysfunction, and lean individuals with unexplained steatohepatitis or advanced fibrosis warrant dedicated consideration for genetic workup [51,52]. As illustrated in Figure 5, a structured decision algorithm can guide clinicians through phenotype-driven testing in this setting, incorporating risk stratification based on variants such as *PNPLA3* rs738409, *TM6SF2* rs58542926, and *HSD17B13*, which have been shown to modulate disease susceptibility and progression. Identifying these variants not only clarifies the underlying disease mechanism but can also influence counseling around lifestyle interventions, pharmacotherapy eligibility, and monitoring intensity [51,52]. Embedding such algorithms within clinical workflows—particularly in patients referred for unexplained liver enzyme elevations or incidentally detected steatosis—represents a practical step toward genomics-integrated *MASLD* care.

**Figure 6:**
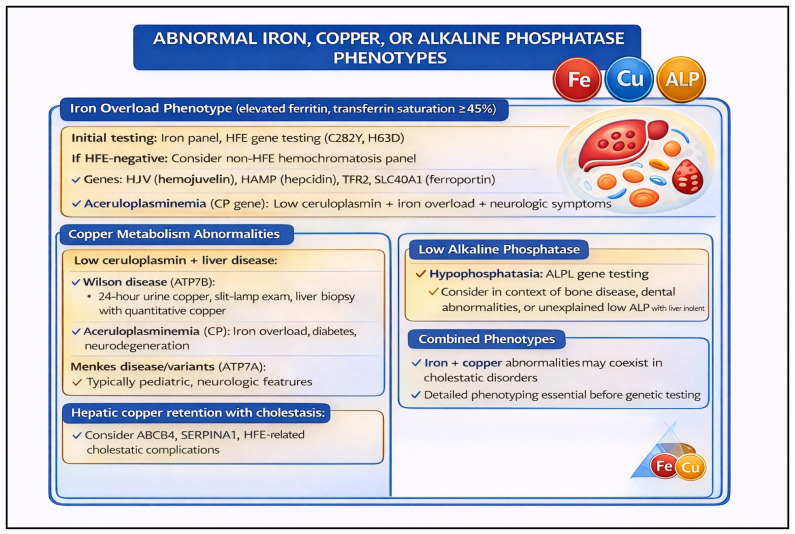
Clinical Decision Tree for Genetic Evaluation in Patients with Unexplained Abnormal Liver Chemistries or Cirrhosis of Unclear Etiology. This algorithm outlines a stepwise approach to genetic testing in adults presenting with persistently abnormal liver enzymes or cirrhosis without an identified cause after standard biochemical and imaging evaluation. Following exclusion of common etiologies per ACG guidelines, the framework stratifies patients by clinical features to guide targeted genetic testing, including hereditary hemochromatosis and other iron overload disorders (e.g., *HFE*, *TFR2*, *HJV*, *HAMP*, *SLC40A1*), as well as rare high-impact variants contributing to cirrhosis susceptibility identified through genome-wide and integrative sequencing approaches. Polygenic risk score integration is highlighted as an emerging adjunct for risk stratification in patients with complex or multifactorial disease.

A final and clinically underappreciated scenario involves patients with persistently abnormal liver chemistries or cirrhosis for which standard evaluation has failed to yield a diagnosis. Figure 6 provides the stepwise testing framework, while the text highlights the key inherited disorders and emerging genomic evidence that justify this approach. This presentation warrants systematic genetic evaluation guided by ACG recommendations for the workup of unexplained liver enzyme elevations [53,54]. Among the most important and actionable genetic disorders to consider in this context are hereditary iron overload syndromes. Hereditary hemochromatosis, caused most commonly by homozygous *HFE* p.C282Y variants but also by rarer pathogenic variants in *TFR2*, *HJV*, *HAMP*, and *SLC40A1*, can present insidiously and is frequently underdiagnosed when iron studies are not interpreted in a genetic framework [55]. Beyond established iron disorders, recent large-scale integrative analyses of common and rare variant data have substantially expanded understanding of the genetic architecture of liver cirrhosis, identifying multiple loci—including both well-established variants (e.g., *PNPLA3*, *TM6SF2*, *HSD17B13*) and novel rare high-impact alleles—that contribute to cirrhosis susceptibility across diverse ancestries [53]. These findings underscore the value of broad genomic evaluation, including panel-based NGS or exome sequencing, in unexplained cirrhosis, and they highlight an emerging role for polygenic risk scores in refining individual risk estimates. Taken together, Figure 6 reinforces the principle that a structured, phenotype-informed genetic workup—rather than ad hoc single-gene testing—is most likely to yield a diagnosis and inform management in this challenging patient population.

#### 3.1.3. Making Results Usable: Integration into the Health Record and Scalable Workflows

Just as important as ordering testing is making results usable. Right now, genetic reports are often stored as non-searchable PDFs in the record, which makes them easy to overlook and difficult to integrate into ongoing care. Barriers to deeper integration include privacy and security concerns, data storage demands, and the variability in how different laboratories return and format results [56]. That said, some institutions that have pursued large-scale integration—such as converting legacy documents into searchable, structured formats within standard laboratory result workflows—have reported early benefits, including improved clinical decision support and more personalized care [57,58]. In the near term, the most effective implementation will likely concentrate in large academic liver centers, given how quickly sequencing technologies and variant-interpretation methods evolve. Over time, as genomic medicine matures and decision-support systems (potentially including artificial intelligence tools) become more widely available, genomic testing should become a practical resource for most hepatologists, not just subspecialty centers.

### 3.2. Development 2: Clinician Readiness and “Genome Rounds” in Hepatology

As genomic information becomes more common in day-to-day care, clinical practice needs to adapt so clinicians can confidently use and interpret genetic results. Broad clinical adoption depends heavily on clinicians feeling comfortable with genomics and understanding how to apply it at the bedside [59]. Even when providers see genetic testing as valuable, many still view it as difficult to use outside research environments [58]. Similar to the interdisciplinary approach encouraged in cardiology for translating genomics into clinical care [60,61], structured “genome rounds” focused on liver disease could accelerate uptake and improve patient care [31].

#### Structure and Purpose of Genome Rounds

We envision recurring multidisciplinary case discussions that include hepatology, clinical genetics, pathology, and other relevant specialists. These sessions would serve two purposes: improving real-time diagnostic decision-making for complex cases, and building durable competence for trainees and practicing clinicians. They would also make it easier to spot atypical phenotypes that suggest a genetic etiology and to standardize when to initiate genomic testing or bring a case forward for group review [62].

### 3.3. Development 3: Scaling Genetic Knowledge—Toward a Liver-Focused Gene Resource

Global sequencing capacity is rapidly expanding, creating unprecedented amounts of genetic data that can be analyzed, reanalyzed, and used for discovery. Multiple existing resources—such as OMIM, ClinVar, HGMD, dbSNP, Clinical Genomics Database, and Orphanet—aggregate variants and gene–disease relationships. However, these databases are manually curated, vary in completeness, and often depend on user submissions, which can introduce inconsistency and delay. Clinical laboratories frequently triangulate across multiple databases to reduce the chance of missing relevant genes [63].

#### 3.3.1. Current Gap: No Single Liver-Focused, Clinician-Friendly, Continuously Updated Database

From a hepatology standpoint, a major gap is the lack of a single, liver-focused, clinician-friendly resource that is consistently updated. Laboratories may use phenotype-driven gene panels or case-specific filtering, but these strategies are not always transparent to the ordering clinician [64]. While compiling liver-related genes from OMIM (as of November 2021 using terms like “hepatic,” “cirrhosis,” and “liver”) can help as a reference, it also illustrates a key limitation: manual curation improves quality control but can lag behind emerging literature, meaning clinically relevant genes may be missing when clinicians need them.

#### 3.3.2. Clinical and Research Value: Diagnosis, Reanalysis Frameworks, and Cross-Center Discovery

A continuously updated liver gene database could directly improve diagnosis for patients who remain unexplained after standard evaluation, especially as the catalog of liver disease genes continues to grow. It would also support a practical framework for reanalysis. At present, there is little guidance on how to perform genetic reanalysis in routine clinical care [64]. Standardized reanalysis approaches could clarify diagnostic yield over time and improve the clinical utility of different sequencing strategies [31]. Genome rounds would reinforce when reanalysis is appropriate, and coordinated collaboration across academic liver centers would help identify shared genotype–phenotype patterns, accelerating recognition of new disorders. Overall, a complete and actively maintained liver-specific gene resource remains an important unmet need for both clinical care and research translation.

### 3.4. Development 4: Genomics-Informed Trial Design and Mechanism Discovery in Common Liver Diseases

Despite major progress in viral hepatitis therapeutics, effective noninvasive treatments remain limited for two of the leading global drivers of chronic liver disease: nonalcoholic fatty liver disease and alcohol-associated liver disease. One reason may be an incomplete understanding of underlying molecular pathways, but another likely contributor is genetic heterogeneity within clinical trial cohorts—where mixtures of risk alleles and protective alleles can obscure true treatment effects or create variable responses across participants. Integrating genotype and molecular diagnosis into trial design and analysis could sharpen the interpretation of outcomes and help align therapeutics with the patient’s biological profile.

#### Rare, High-Impact Variants as a Path to Causal Mechanisms and Targets

In addition, identifying genes that carry rare, high-impact deleterious variants offers a powerful way to connect genotype to phenotype and establish causal mechanisms. These rare pathogenic variants can also reveal therapeutic targets that might otherwise be missed, opening new avenues for noninvasive therapy development [65].

### 3.5. Development 5: Genetic Medicines, Molecular Diagnosis, and the Shift Toward Multiomics

As genomics becomes routine across both clinical care and research, opportunities expand for therapies that directly modify gene expression or correct molecular defects. These “genetic medicines” may involve gene addition, gene editing, gene silencing, or transient expression approaches using DNA or messenger RNA platforms [65]. The hepatic tropism of both viral and lipid-nanoparticle (LNP) delivery systems—a direct consequence of the sinusoidal architecture and fenestrated endothelium described in Section 2—has made the liver one of the most tractable organs for genetic medicine, and several therapies have already completed the transition from bench to bedside. In the hemophilia space, two AAV-based gene therapies have received regulatory approval: etranacogene dezaparvovec (Hemgenix; uniQure/CSL Behring), an AAV5 vector encoding a high-activity factor IX variant for haemophilia B, was approved by the FDA in November 2022 and demonstrated sustained factor IX activity that eliminated most treated patients’ annualized bleeding rate across a pivotal trial [66]; and valoctocogene roxaparvovec (Roctavian; BioMarin), an AAV5 vector for haemophilia A, received FDA approval in June 2023 following evidence of marked reduction in factor VIII supplementation requirements [67]. Beyond haemophilia, hepatically delivered RNA-based therapies have established an independent clinical track record. Patisiran (Onpattro; Alnylam), an siRNA formulated in an LNP, was the first RNA interference therapeutic approved anywhere (FDA, 2018) and targets TTR mRNA in hepatocytes to treat transthyretin amyloidosis with polyneuropathy; it has since been joined by givosiran (Givlaari), an siRNA licensed for acute hepatic porphyria [68,69]. Most recently, inclisiran (Leqvio; Novartis), a twice-yearly subcutaneous siRNA/LNP directed at PCSK9 mRNA in the liver, has been approved for LDL-lowering, illustrating how hepatic RNA silencing has entered mainstream cardiovascular medicine [70]. Given the real-world demonstration of safe and effective mRNA vaccines during the COVID-19 pandemic [71,72], it is reasonable to expect broader application of mRNA-based therapeutics across medicine, including hepatology. Over time, hepatology practice is likely to become increasingly organized around molecular diagnosis—understanding why a given patient develops liver disease at the pathway level—so that treatment can be individualized and mechanism-based.

### 3.6. Beyond Genomics: Multiomics and Real-World Discovery

Finally, while this discussion centers on genomics, the broader direction of the field is moving toward multiomics: integrating genomics with transcriptomics, proteomics, epigenomics, and metabolomics, alongside careful phenotyping and environmental or medication exposures. This kind of multimodal analysis is well-positioned to uncover new genotypes–phenotype relationships, biomarkers, and therapeutic targets. Large-scale electronic health record–linked studies have already been used to identify both risk and protective variants in liver disease [73]. Taken together, these trends point toward a hepatology landscape where molecular diagnostics and targeted, noninvasive therapies become central—ideally slowing fibrosis progression and reducing the need for liver transplantation.

## 4. Conclusions

The liver’s unique anatomy and immune architecture make it both a biologically attractive target and a biologically selective barrier for genomic medicine. In this review, we argue that these features are not merely background physiology; they are central determinants of how genomic diagnostics, delivery platforms, and gene-based therapies perform in hepatology. Framed in this way, earlier genomic testing, multidisciplinary genome rounds, a centralized liver-specific gene resource, genotype-informed trial design, and the expansion of genetic medicines represent not separate trends, but interconnected components of a genomics-driven hepatology model. Realizing this model will require continued improvement in clinician education, evidence-based implementation guidance, health record integration, and equitable access to genomic tools. Looking ahead, integration of genomics with transcriptomics, proteomics, metabolomics, and real-world clinical data may further refine molecular diagnosis, risk stratification, and individualized therapy across the spectrum of liver disease.

## Data Availability

Not applicable.

## Abbreviations

ACG	American College of Gastroenterology
ACMG	American College of Medical Genetics and Genomics
AGA	American Gastroenterological Association
AASLD	American Association for the Study of Liver Diseases
EASL	European Association for the Study of the Liver
DNA	deoxyribonucleic acid
RNA	ribonucleic acid
mRNA	messenger ribonucleic acid
NGS	next-generation sequencing
GWAS	genome-wide association study
PRS	polygenic risk score
*MASLD*	metabolic dysfunction–associated steatotic liver disease
NAFLD	nonalcoholic fatty liver disease
HCC	hepatocellular carcinoma
PALF	pediatric acute liver failure
LSEC(s)	liver sinusoidal endothelial cell(s)
HSC(s)	hepatic stellate cell(s)
ECM	extracellular matrix
EMR	electronic medical record
EHR	electronic health record
IL	interleukin (e.g., IL-1β, IL-6, IL-8, IL-12)
TNF-α	tumor necrosis factor alpha
TGF-β/TGF-β1	transforming growth factor beta/transforming growth factor beta 1
CD8+	cluster of differentiation 8 positive (T cells)
NK	natural killer (cells)
NKT	natural killer T (cells)
MAIT	mucosal-associated invariant T (cells)
γδ T cells	gamma delta T cells
HBV	hepatitis B virus
HCV	hepatitis C virus
OMIM	Online Mendelian Inheritance in Man
HGMD	Human Gene Mutation Database
dbSNP	Single Nucleotide Polymorphism database (dbSNP)
